# Identification of individual-level clinical factors associated with increased risk of death during heatwaves: a time-stratified case-crossover study using national primary care records in England

**DOI:** 10.1136/bmjph-2024-000927

**Published:** 2024-05-27

**Authors:** Ross Thompson, Sari Kovats, Shakoor Hajat, Helen Macintyre, Emer O’Connell

**Affiliations:** 1 NIHR Health Protection Research Unit in Environmental Change and Health, London School of Hygiene & Tropical Medicine, London, UK; 2 Extreme Events and Health Protection, Centre for Climate and Health Security, UK Health Security Agency, London, UK; 3 Climate and Health Assessment Team, Centre for Climate and Health Security, UK Health Security Agency, London, UK; 4 School of Geography, Earth and Environmental Sciences, University of Birmingham, Birmingham, UK

**Keywords:** Public Health, Epidemiology, Environmental Monitoring

## Abstract

**Background:**

Despite an increase in heat-related deaths occurring in England in recent years, one of the key recommended actions of identifying individuals at risk and deploying targeted interventions is not routinely undertaken. A major contributing factor to this is a lack of understanding of the individual-level risk factors that would support an evidence-based approach to targeted prevention.

**Objective:**

To identify individual-level clinical risk factors for heat-related mortality in England by using primary care records and to estimate potential effect modification of a range of pre-existing conditions, clinical measurements and prescribed medications.

**Methods:**

A time-stratified case-crossover analysis was undertaken of 37 individual-level clinical risk factors. Patient’s data were obtained from the Clinical Practice Research Datalink. Conditional logistic regression was used to characterise associations between temperature and the risk of death on hot days.

**Results:**

Heat mortality risk was modified by a large range of pre-existing conditions, with cardiorespiratory, mental health and cognitive function conditions, diabetes and Parkinson’s, all increasing risk. The most striking increase was observed for depression with an OR of 1.25 (95% CI 1.09 to 1.44), the highest observed for pre-existing conditions. Individuals prescribed medications to treat heart failure and high blood pressure also have increased odds of death during heatwaves. There appears to be evidence of an increasing trend in ORs for diastolic blood pressure (DBP) categories, with ORs increasing from low DBP up to prehypertensive DBP group.

**Conclusions:**

This is the first study to explore a comprehensive set of individual-level clinical risk factors and heat using primary care records in England. Results presented have important implications for patient medication management during heat events, incorporating heat-risk considerations into other health policies such as suicide prevention plans and highlighted potential differences between clinical vulnerability and patients at risk.

**WHAT IS ALREADY KNOWN ON THIS TOPIC**

- Previous epidemiological studies exploring individual-level heat risk factors have generally used routine mortality and emergency hospitalisation data to characterise heat risks in persons with pre-existing conditions. This has limited use to support primary care clinicians in protecting their patients as they focus only on severe disease and provide no intelligence on risk associated with ongoing treatment or medication use.

**WHAT THIS STUDY ADDS**

- This is the first study to explore individual-level clinical risk factors in England using primary care records.

**HOW THIS STUDY MIGHT AFFECT RESEARCH, PRACTICE OR POLICY**

- We have demonstrated that primary care data can provide powerful insights that have implications for how clinicians prioritise patients during heatwaves, manage patient medication and implications for other health policy areas such as suicide prevention.

## Introduction

 Heatwaves pose a significant risk to health.^1^,^4^ In England, heat-related deaths have been increasing since 2016.^5^ In summer 2022, England experienced its first 40°C heatwave and associated level 4 Heat-Health Alert, resulting in 2985 excess deaths.^6^ Following the major European heatwave in 2003, many countries introduced Heat-Health Actions Plans (HHAPs), which outline a framework for the health sector to respond to these events.^7^ The UK Health Security Agency (UKHSA) launched the revised HHAP for England, the Adverse Weather and Health Plan (AWHP) in 2023 which aims to prevent avoidable harm to health during periods of adverse weather, including extreme heat. A key action for health and social care providers recommended in the AWHP is to ‘establish methods to identify, alert and monitor individuals most vulnerable to heat-related illnesses on your caseload’.^8 9^ This recommendation is not widely implemented^10 11^ and one reason is the absence of an evidence-based process through which healthcare professionals can identify individuals most at risk of dying in a heatwave.^11 12^

The pathophysiological mechanisms of ill health during periods of heat generally result from impaired thermoregulatory (eg, vascular dilation and sweating) or behavioural responses.^13 14^ At the population level, subgroups identified as at risk include older adults, children, those with chronic conditions, including mental health conditions, those prescribed certain medication and those unable to adapt their own behaviours or environments.^5 15^ Epidemiological studies exploring individual heat risk factors have generally used routine mortality and emergency hospitalisation data to characterise important chronic conditions.^16^,^20^ However, these studies provide limited evidence to support action and clinical decisions. Results from these studies are not specific to the English population leading to uncertainty about the generalisability of the findings. The use of emergency admissions data may not account for individuals receiving treatment or care within the community who do not enter the hospital system prior to death so these studies are likely biased towards individuals with more severe disease. This is especially relevant as a large proportion of deaths during heatwaves occur in the home.^21^ Nor do they include information about ongoing treatment, such as prescribed medication. All of these may play a significant role in an individual’s overall risk and may underestimate the effects of these risk factors.

Therefore, the aim of this study is to identify individual-level clinical risk factors for heat-related mortality in England by using primary care records and to estimate the potential effect modification of a range of pre-existing conditions, clinical measurements and prescribed medications. Results from this study provide foundational evidence on which methodologies and processes can be established to help healthcare professionals in identifying individuals most at risk so that targeted interventions can be deployed.

## Methods

### Study population

We used the Clinical Practice Research Datalink (CPRD) Aurum (ID number 21_000621) to link primary care records, Office for National Statistics (ONS) mortality data and National Health Service hospitalisation data, at the individual level. The outcome was defined as all deaths which occurred between May 2016 and September 2020 using ONS date of death. CPRD Aurum has been shown to be representative of the English population.^22^

Primary care records were used to identify individuals with pre-existing disease, prescribed medication and blood pressure measurements. Records were valid if they were recorded within 2 years of death to better reflect a diagnosis at the time of death (ie, records between 2014 and 2020).^16 17^ Where there was more than one relevant record within the 2-year window, the record closest to the date of death was used. Pre-existing conditions and prescribed medication groups were selected a priori based on published evidence, physiological plausibility and data availability as presented in online supplemental file S1. This resulted in a total of 37 priority variables identified (see table 1). Age and gender were not included in this analysis, however, will be in further analysis exploring wider determinants of health. A combination of bespoke and published clinical code lists were used to create risk factor variables for all individuals in the study sample. Clinical code lists are available in online supplemental table S1.

**Table 1:** Overview of data used in analysis with a total number of individuals with a valid primary care record that occurred within 2 years of death by subnational region, pre-existing condition, prescribed medication and blood pressure measurements

Variable	Individuals	Proportion
All persons	430 682	100.00%
Subnational regions	430 682	100.00%
The North (NE, NW and Y&H)	113 405	26.33%
Midlands and East (WM, EM, EoE)	102 630	23.83%
London	65 145	15.13%
The South (SW and SE)	149 502	34.71%
Diastolic blood pressure	232 518	53.99%
Low (<60 mm Hg)	25 416	5.90%
Normal (60–79 mm Hg)	142 421	33.07%
Prehypertensive (80–89 mm Hg)	49 781	11.56%
Hypertension Stage 1 (90–99 mm Hg)	11 588	2.69%
Hypertension Stage 2 (100 mm Hg+)	3336	0.77%
Hypertension (1 and 2)	14 924	3.47%
Systolic blood pressure	232 785	54.05%
Low (<80 mm Hg)	1442	0.33%
Normal (80–119 mm Hg)	80 680	18.73%
Prehypertensive (120–139 mm Hg)	96 646	22.44%
Hypertension stage 1 (140–159 mm Hg)	43 418	10.08%
Hypertension stage 2 (160 mm Hg+)	10 623	2.47%
Hypertension (1 and 2)	54 041	12.55%
Alzheimer’s and dementia	34 610	8.04%
Anxiety	11 343	2.63%
Arrythmia	32 279	7.49%
Asthma	9118	2.12%
Bipolar disorder	726	0.17%
Cardiac arrest	2084	0.48%
Cardiomyopathy	1148	0.27%
Chronic kidney disease	25 252	5.86%
Chronic obstructive pulmonary disease	11 655	2.71%
Depression	5705	1.32%
Diabetes	48 391	11.24%
Emphysema	1847	0.43%
Haemorrhage	3222	0.75%
Heart failure	22 345	5.19%
Hyperthyroidism	718	0.17%
Hypothyroidism	7908	1.84%
Severe learning disability	1135	0.26%
Chronic liver disease	3738	0.87%
Myocardial infarction	7338	1.70%
Occlusion	5841	1.36%
Other cerebrovascular diseases	1407	0.33%
Parkinson’s disease	4970	1.15%
Psychosis	6105	1.42%
Schizophrenia	942	0.22%
Severe mental illness	2412	0.56%
Stroke	11 736	2.72%
Prescribed medication		
Ace inhibitors	64 959	15.08%
Beta blockers	59 996	13.93%
Cardio glycols	12 136	2.82%
Diuretics	71 262	16.55%
Non-steroidal anti-inflammatory drugs	49 020	11.38%
Vasoconstrictor drugs	547	0.13%
Anticholinergic drugs	8952	2.08%
Total number of practices contributing to sample=1476Mean number of patients per practice=291.79		
Maximum number of patients per practice=2400		
Minimum number of patients per practice=6		

Severe mental health is a composite indicator of severe mental health conditions including schizophrenia, bipolar disorder, manic episodes, etc. Clinical code list available in online supplemental materials.

Summary statistics also provided for continuous variables which were categorised for analysis.

EMEast MidlandsEoEEast of EnglandNENortheastNWNorthwestSESoutheastSWSouthwestWMWest MidlandsY&HYorkshire and the Humber

### Exposure data

Geographical information on individuals in CPRD is limited to the UK government region of their registered primary care practice. Therefore, a daily mean population-weighted regional temperature series was generated for the study period (May 2016–September 2020). Daily mean temperatures were generated from HadUK-grid daily maximum and minimum temperatures.^23 24^ The gridded mean daily temperature series was then combined with 100 m gridded population data using ArcGIS to create regional population-weighted temperature series which were then assigned to each individual based on their general practitioner (GP) practice region. A lag period of 0–2 (3 days) were also calculated and assigned to each individual to estimate delayed and cumulative effects of exposure over the 3 days. Heat effects are known to be mostly immediate so impacts at longer lags were not considered.^25^

### Statistical analysis

A time-stratified case-crossover study design was employed to assess the association between temperature and mortality. Within this study design, the temperature on the day of death (event-day) is compared with non-event days. The main relationship under investigation is the association between temperature and risk of death on days above specified temperature thresholds using conditional logistic regression. The case serves as its own control and therefore the potential effect of time-independent confounding factors such as age or gender are automatically controlled for. Case days were determined as the date of death. Control days were selected following a bidirectional referent selection approach, to be the same day of the week of the same month in which the death occurred, resulting in each case having at least three controls, reducing the potential for overlap bias.^26^ This is a popular and robust approach used within environmental epidemiology for estimating the association of acute health events in response to short-term exposures.^16 17 27 28^

First the association between temperature and mortality was modelled to assess the dose–response relationship between temperature and risk of death. This was carried out using natural cubic spline functions, with internal knots determined using the Akaike information criterion to define the best model fit. From this initial model, temperature thresholds for analysis were derived. To maximise policy relevance, thresholds were selected using the approach used by the UKHSA for defining the ‘low’ impact level of the new impact-based Heat-Health Alert system.^29^ That is, the temperature is associated with a relative risk (RR) of 1.1, that is, a 10% elevated risk of death. The ‘medium’ impact threshold, defined as the temperature associated with an RR of 1.2, was used in sensitivity analysis. The ‘high’ impact threshold was not used in this analysis due to daily temperatures within the study period not reaching the required 40°C temperatures, as defined by UKHSA. Relative thresholds were derived for the national-level analysis and for subnational-level analysis, also carried out as sensitivity analysis. The reference temperature for the conditional logistic regression was taken as the minimum mortality temperature (MMT) which is the temperature at which risk of death is lowest. Results were stratified by condition/medication to assess effect modification.

All results are reported as ORs with 95% CIs and p values. In addition, to aid in the assessment of the potential modifying effect of each subgroup, a relative effect modification (REM) index was calculated as the specific OR of an individual-level factor compared with a reference category. For categorical factors, such as blood pressure, the reference category was taken as ‘normal blood pressure’. For all binary variables, however, the reference category was taken as the OR estimate for the whole population. All analyses were carried out in Stata Statistical Software: release V.17.^30^

### Sensitivity analysis

First, the analysis was repeated using the ‘medium impact’ threshold to assess any differences in the patterns of ORs. Second, the analysis was again repeated at subnational level to assess geographical variations in estimated associations. Subnational-level analysis was carried out using the following regional groups: London; The North (combined North East, North West and Yorkshire and Humber); Midlands and East (combined West Midlands, East Midlands and East of England) and the South (South West and South East). Regions were combined to ensure that the frequency of the events (deaths, heat-health alerts) was sufficient to support the analysis and based on study population frequencies, the study population distribution compared with the national distribution over the study period, the number of Heat-Health Alerts issued over the study period, geographical location and climate.

Third, the analysis was carried out using hospitalisation data to define pre-existing disease status. This was to assess any significant differences in the effect sizes observed between the two approaches to defining pre-existing conditions. This was carried out for a limited number of conditions which had strong evidence for a significant association within the main analysis.

And finally, we assessed the potential confounding effect of background air pollutants on a restricted number of pre-existing conditions and medications. Particulate matter (PM_10_), ozone (O_3_) and nitrogen dioxide (NO_2_) are all potential confounders in the association of mortality and heatwaves.^31^ Due to data limitations, the analysis was restricted to London and daily mean NO_2_, PM_10_ and O_3_ concentration values. Five urban background air quality monitoring sights were selected across London from the London Air Quality Network.^32^ Using daily mean values for each site, a London-wide daily mean background value was derived for each pollutant and assigned to each individual in London. As with temperature, a lag period of 0–2 days was also calculated and assigned to each individual.

### Patient and public involvement

The Health Protection Research Unit in Environmental Change and Health has developed a public engagement/involvement group called PLANET, which was established in Autumn of 2020. PLANET stands for Public Led and Knowledge Engagement Team. The group has 30 members and approximately 20 attend the regular meetings every 3–4 months to discuss research projects. A session exploring heat risk and the use of primary care records to identify those at highest risk was carried out in May 2022 where the aims and objectives were presented to the group for feedback. Results from this study will be presented to the PLANET group in early 2024 at an annual meeting, with the meaning and implications of the results discussed, and potential future research in this area explored.

## Results

430 682 individuals who died during the study period between May 2016 and September 2020, from 1476 primary care practices were included in the study population. A full breakdown of the numbers of individuals with suitable records for each individual-level factor considered within this analysis is detailed in table 1. The study population age profile indicates that it is heavily skewed towards the older population and is aligned with the national age distribution. Details of exposure data are available in online supplemental table S2.

### Temperature thresholds

**Figure 1:**
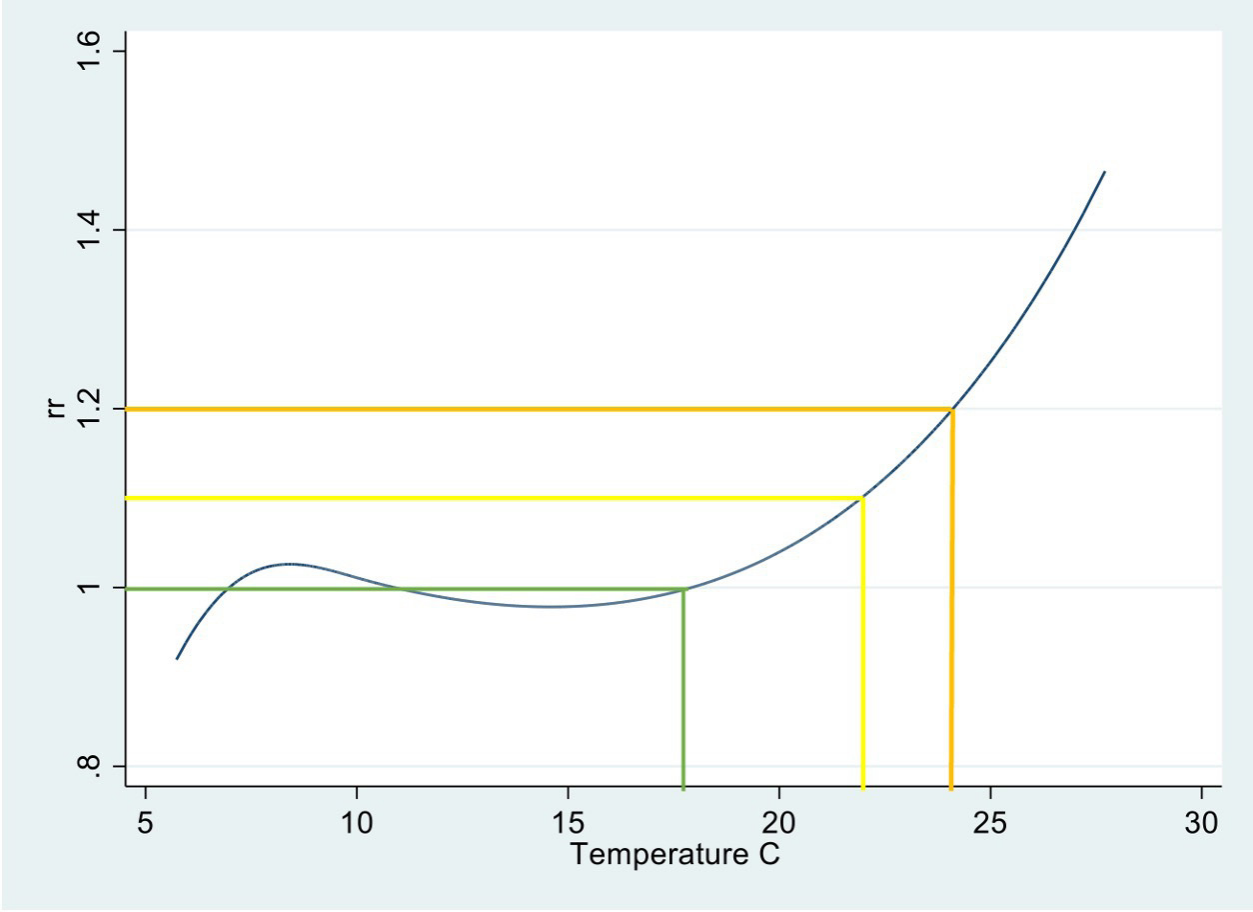
National temperature-mortality relationship plot of relative risk (RR) and mean temperature from which temperature thresholds used in analysis were derived. Green line represents the MMT with an RR=1.0 which equates to about 17°C (rounded to the nearest 0.5°); yellow line indicates the UKHSA defined low impact threshold with an RR of 1.1 which equates to 22°C; amber lines indicate the UKHSA defined Medium Impact threshold with an RR of 1.2 which equates to 24°C which was used in sensitivity analysis. MMT, minimum mortality temperature. UKHSA, UK Health Security Agency

Figure 1 shows the temperature–mortality relationship derived using the full data series (ie, all individuals within the study population) and the temperature thresholds derived. The policy-relevant thresholds, when rounded to the nearest 0.5°C equate to 17°C (the MMT), 22°C and 24°C. Using these thresholds to identify cases resulted in 13 970 using the ‘low’ impact threshold and 10 187 using the ‘medium’ impact threshold. Temperature thresholds used for subnational-level sensitivity analysis are reported in online supplemental table S3.

### Pre-existing conditions

**Figure 2:**
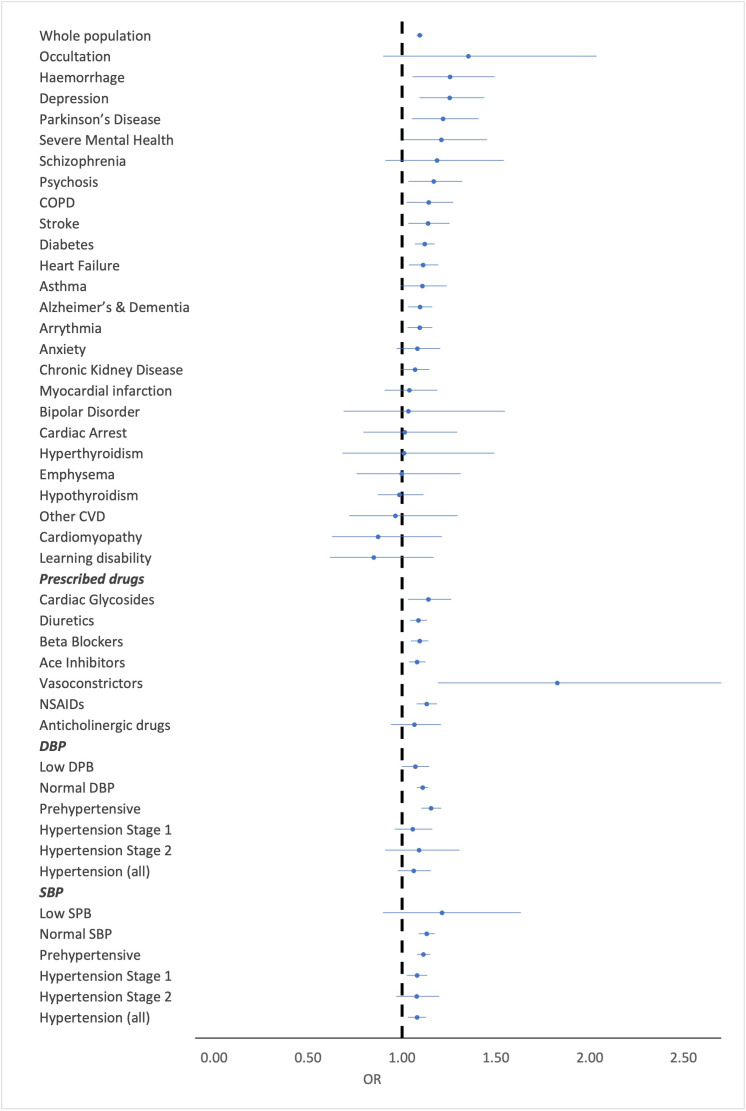
Forest plot of OR and 95% CI for all chronic conditions, prescribed medication groups and results for diastolic and systolic blood pressure categories. COPD, chronic obstructive pulmonary disease; DBP, diastolic blood pressure; SBP, systolic blood pressure; CVD, cardiovascular diseases.

Heat mortality risk was modified by a large range of pre-existing conditions when comparing the odds of death at the MMT (17°C) and ‘low impact’ temperature (22°C) as illustrated in figure 2, with cardiorespiratory conditions all increasing risk. Most striking, however, is that 4 out of 12 conditions with strong evidence (p<0.01) of an association are mental health or cognitive function conditions, including depression, psychosis, severe mental health conditions and Alzheimer’s and dementia. Depression also had the largest relative modification effect of all pre-existing conditions with an REM index value of 1.15 compared with the whole population, however, 95%CIs overlap. Diabetes and Parkinson’s disease were also found to modify risk of death during periods of heat.

### Prescribed medication

Individuals prescribed medications to treat heart failure and high blood pressure had increased odds of death during heat episodes (22°C+) compared with non-heat days (<17°C). The point estimate for patients prescribed vasodilators is relatively high, at 1.83 (1.19 to 2.80), with large 95% CIs due to the small number of individuals in this subgroup. There was also strong evidence that non-steroidal anti-inflammatory drugs increase the odds of death during heatwaves. Evidence for an association with anticholinergic drugs was weak, although the point estimate is comparable to other similar medication classes investigated.

### Blood pressure

There appears to be evidence of an increasing trend in ORs for diastolic blood pressure (DBP), with ORs increasing from low DBP up to prehypertensive DBP. However, this trend is not apparent in patients with hypertension 1 and 2 groups, where ORs reduce considerably. The opposite trend is observed for systolic blood pressure (SBP) categories with a decreasing trend in OR estimates with each increasing category of SBP (figure 2).

### Sensitivity analysis 1: ‘medium’ impact threshold and subnational-level analysis

For the first sensitivity analysis, in general, the patterns observed in the study population at national level were mirrored at subnational level. However, some additional conditions displayed strong evidence for an association at subnational level that were not evident at the national level (see online supplemental table S4). Similarly, patterns in ORs reported here were replicated when using the higher, medium impact temperature thresholds. Full results are available in online supplemental table S5.

### Sensitivity analysis 2: use of emergency admissions data to define pre-existing disease

**Figure 3:**
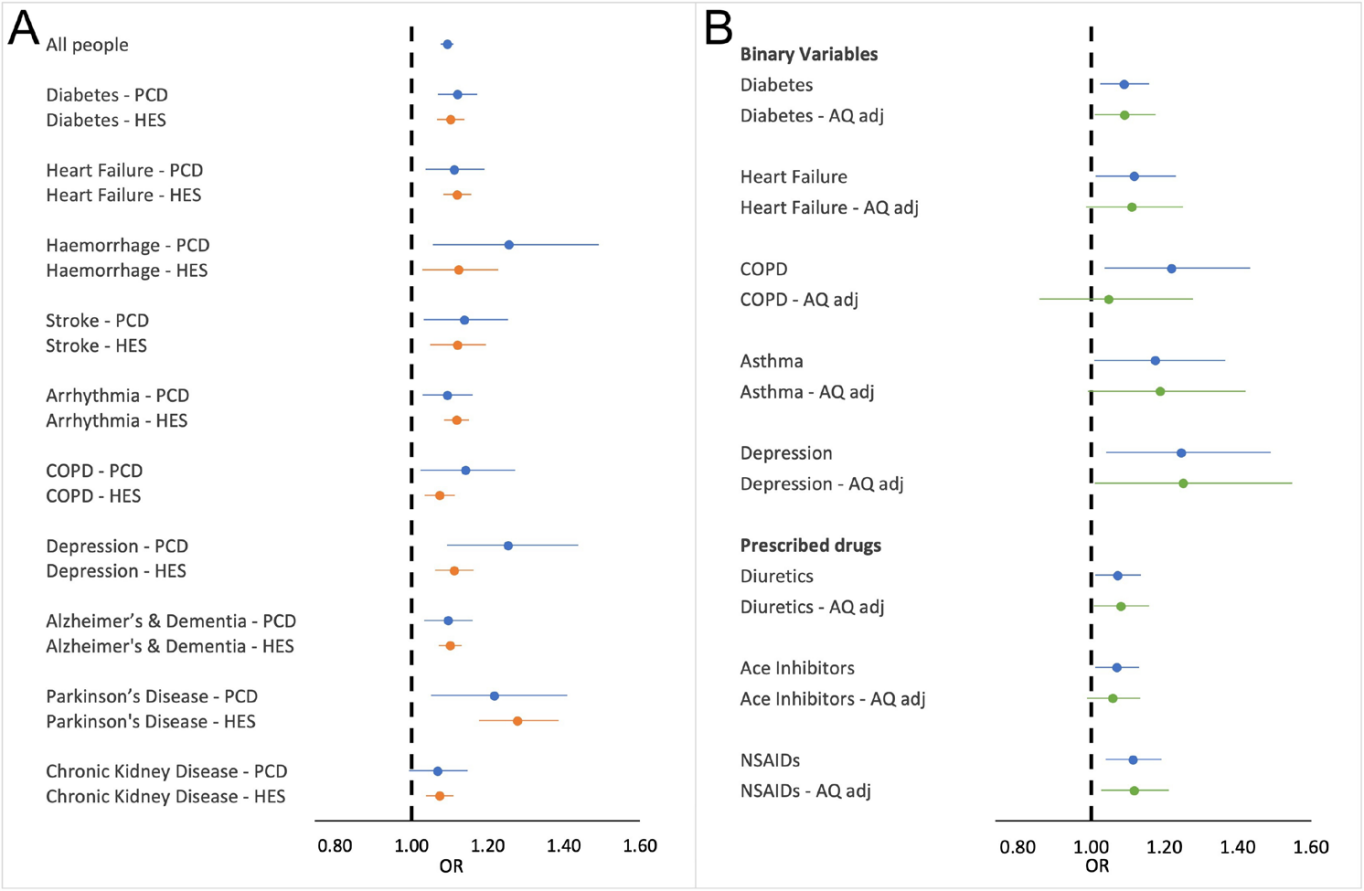
Sensitivity analysis forest plots. (A) The ORs and 95%CI of death on hot day for chronic conditions defined using primary care data (PCD) indicated in blue compared to those same conditions as defined using HES admitted patient care emergency admissions data (HES), indicated as orange. (B) Unadjusted ORs and 95%CI of death on a hot day in London (blue) and the OR and 95%CI estimates adjusting for background daily mean concentrations ozone, nitrogen dioxide and PM10 in London (green). COPD, chronic obstructive pulmonary disease; NSAIDs, non-steroidal anti-inflammatory drugs; PM10, particulate matter; HES, hospital episode statistics.

Overall, ORs were markedly consistent across the two data sources used to define the conditions investigated (figure 3A). A number of the conditions are associated with higher ORs using GP data than with emergency hospitalisation data, such as haemorrhage, CPOD and particularly depression; however, the 95% CIs for both data sources overlap.

### Sensitivity analysis 3: adjusting for background air quality indicators in London

The air-quality-adjusted estimates for all variables were consistent across models. Figure 3B provides comparison of variables with strong evidence of an association between increased odds of death during periods of heat in the unadjusted model and the adjusted model. The adjusted and unadjusted OR estimates are broadly similar for all conditions apart from chronic obstructive pulmonary disease (COPD) which has a considerably reduced OR estimate when adjusted for air pollutant concentrations.

## Discussion

To our knowledge, this study is the first to explore individual-level risk factors associated with heatwaves as recorded within primary care records in England. This study has shown very clearly that risk of mortality increases during periods of heat for individuals with a range of pre-existing disease including cardiorespiratory conditions, mental health and cognitive function conditions, diabetes and Parkinson’s disease, with the largest increases observed for those with a record of depression and haemorrhage in the 2 years proceeding death. In addition, we also demonstrate that individuals prescribed non-steroidal anti-inflammatory drugs (NSAIDs) and medications used to treat high blood pressure and heart failure are also at increased risk of death during periods of heat. We have explored the role of air pollution as a concurrent risk, providing evidence that it may be the dominant exposure of concern for patients with COPD but may be less important for other patient groups affected by heat exposure. Finally, we have identified an unexpected pattern in risk by DBP groups.

The observed patterns in ORs by individual-level clinical risk factors investigated were remarkably consistent across each sensitivity analysis. It appears our results are unlikely to be confounded by air pollution concentrations, except for COPD. When we compared our results using primary care data to those defined by emergency hospitalisation data, ORs were again very consistent, however, estimates were higher for some individual-level factors defined by primary care records. This suggests that for some pre-existing conditions which are more routinely treated within primary care, primary care records may be more sensitive to identifying a signal for heat risk.

Some findings align with population-level evidence within the broader literature, specifically that odds of death for individuals with circulatory and respiratory diseases is increased during heatwaves.^15 16 33^ However, one unexpected observation was that individuals classed as hypertensive (DBP) did not follow the overall trend of increasing ORs with increasing DBP group. This contradicts previous studies that suggest this group may be at increased risk.^34^ While physiological pathways suggest this group is potentially more vulnerable to the effects of high temperatures, our results raise questions about potential differences in physiological vulnerability and those actually at-risk during heatwaves. One plausible explanation may be linked to the management of hypertension via clinical interventions. This would align with evidence observed elsewhere suggesting that the level of care received by individuals, regardless of high temperatures, may reduce heat risk in individuals receiving treatment.^17^ This potentially has important implications for how clinicians prioritise patients in their care during heat events who might not be the most physiologically vulnerable but at the highest level of risk.

One of the most striking observations was the association between mental health conditions and increased odds of death during heat, with depression particularly standing out. This not only has implications for HHAPs but also extends to suicide prevention strategies, given the link between depression as a risk factor for suicide and the strong evidence for increased risk of suicide during periods of high temperature.^35^ Our finding that individuals with Alzheimer’s and dementia, Parkinson’s disease and diabetes are at risk during heatwaves also aligns with recent population-level research.^28 33 36^ These conditions are all associated with older age and emphasise the need for targeted and efficient responses as the number and proportion of older people in our population grows.^37^ Integrating heat-risk considerations into broader health agendas, especially within the context of evolving person-focused care in community models, is becoming crucial.

As with the existing literature, our finding of increased risk for individuals prescribed medications for heart failure or blood pressure^38^ underscores the need for evidence-based individually-tailored medicine management with presummer reviews to reduce risk during heatwaves. Currently, we are unaware of any guidance for clinicians or patients which addresses this need. Additionally, our finding that NSAIDs appear to increase risk during periods of heat suggests there is a need for evidenced-based clinical guidance beyond just cardiovascular drugs. However, due to the diversity in drug types and modes of action, we could not explore all potential medications that could increase heat-related risks. This is a priority area that requires further research.

### Limitations of the study

Geographical resolution of the health data limits precise exposure assignment for each individual within the study. However, previous studies^1^ have demonstrated the high correlation between temperature monitoring stations within English regions, and that it is possible to characterise exposure well using a regionally representative temperature series. In addition, inconsistencies in primary care consultation records and the specific terms used when recording the details by clinicians increase the potential for some relevant records to be missed. However, a systematic approach for identifying relevant records which included clinical validation was used and should address this limitation. While CPRD is representative of the English population^22^ it does not have full coverage of England and is not geographically representative.^22^ Episode analysis of previous heatwaves suggests most heatwave-related deaths occur in the south.^639^,^44^ Therefore, it is not anticipated that this limitation would affect our results significantly. Within this analysis, it was not possible to investigate the potential multiple interactions between different individual-level risk factors, and how this may modify an individual’s overall risk. Nor did we investigate wider determinants of health which may be recorded within primary care data, that might provide additional intelligence on who is most at risk. While it is well documented that the older population are at increased risk, the focus of this study was to look at specific clinical and diagnostic criteria recorded within primary care records. These additional elements are critical areas which require further research.

## Conclusions

This is the first study to explore individual-level clinical risk factors and heat using primary care records in England and has highlighted important factors associated with increased risk of death during heatwaves. We have demonstrated that primary care data can provide useful insights into important differences between those who might be characterised as clinically vulnerable and those who are at greater risk. Results from this study suggest implications beyond HHAPs alone but transcend into other health policy areas, for example, suicide prevention plans and healthy ageing. In addition, more research is needed on the role that a wide range of medication use potentially has in modifying heat risk. Research is also required to explore other factors recorded within primary care data to gain further insight into heat risk at the individual level. This further intelligence could then be combined with insights from this study to develop an evidenced-based approach to identifying individuals most at risk within the community so that targeted interventions can be deployed.

## supplementary material

10.1136/bmjph-2024-000927 online supplemental file 1

## Data Availability

Data may be obtained from a third party and are not publicly available.
