# Supplementary material for: Identification of individual-level clinical factors associated with increased risk of death during heatwaves: a time-stratified case-crossover study using national primary care records in England
Source: BMJ Public Health. 2024 May 27;2(1):e000927. doi: 10.1136/bmjph-2024-000927 (PMC11812907; doi:10.1136/bmjph-2024-000927)
Supplement: online supplemental file 1 [file bmjph-2-1-s001.pdf]

Using individual-level clinical factors and prescribed medicines to identify those at risk of death during heatwaves – a time-stratified case-crossover study using national primary care records –
<u>Supplementary materials</u>

Authors:
Ross Thompson[1,2], Sari Kovats[1], Shakoor Hajat[1], Helen Macintyre[3,4], Emer O'Connell[1,5]

Affiliations:
[1] NIHR Health Protection Research Unit in Environmental Change and Health, London School of Hygiene and Tropical Medicine, London
[2]Extreme Events and Health Protection Team, UK Health Security Agency, London
[3]Climate and Health Assessment Team, Centre for Climate and Health Security, UK Health Security Agency
[4]School of Geography, Earth and Environmental Sciences, University of Birmingham
[5] Public Health Unit, Greater London Authority

Contents

Table S1 - Justification for inclusion of individual level variables, definition of variable and source of clinical codes
Table S2. Description of exposure data series in analysis.
Table S3 Temperature thresholds used in sub-national analysis derived from the temperature mortality relationships.
Table S4 - Results for national and sub-national level unadjusted odds ratios for individuals with pre-existing conditions and prescribed medication with very strong to moderate evidence of increased risk of death during periods of heat using the low impact threshold compared to the minimum mortality temperature and the relative effect modification index (REM).
Table S5 – Full results across both low and high impact temperature thresholds

Table S1. Table outlines each variable included in the analysis, justification for inclusion and information about how each was defined and link to clinical code lists used to extract the data.

**Variable for analysis**	**Hypothesis to be tested and likely mechanisms**	**Definition of variable**	**Reference for CPRD Aurum code list(s) used to define variables of interest**
Diabetes	Diabetes increases an individual's risk of death during periods of heat, either directly or indirectly.<br><br>*Likely mechanism*: diabetes is a recognised risk factor for a range of conditions which are also known to increase risk of mortality during heatwaves, e.g. cardiovascular diseases. Therefore, diabetes may indirectly increase risk.	Diagnosis of type 1 and type 2 diabetes within the two years before death. Binary variable (yes or no).	Davidson, J, Warren-Gash, C, Mcdonald, H, Banerjee, A, Smeeth, L, Evans, D and Clay, S. Clinical codelist - Diabetes [Internet]. London School of Hygiene & Tropical Medicine; 2021. Available from: https://doi.org/10.17037/DATA.00002407.
Myocardial infarction	Experiencing a heart attack increases an individual's risk of death during periods of heat<br><br>*Likely mechanism:* increased strain on heart to maintain blood pressure due to increased viscosity of blood	Diagnosis/record of myocardial infarction (including subsequent MI) within the two years before death. Binary variable (yes or no).	Nealon J, Modin D, Ghosh RE, et al. The feasibility of pragmatic influenza vaccine randomized controlled real-world trials in Denmark and England. NPJ Vaccines 2022;7(1) doi: https://doi.org/10.1038/s41541-022-00444-6<br><br>A range cardiovascular disease CPRD Aurum specific code lists, mapped to ICD-10 categories are available. To capture all MI cases the following code lists were used in combination:<br>• *Myocardial infarction (ICD-10: I21)*<br>• *Subsequent myocardial infarction (ICD-10: I22)*
Cardiomyopathy	High temperatures increase risk of death for individuals who suffer from conditions that affect the hearts' ability to pump blood.	Diagnosis/record of cardiomyopathy within the two years before death. Binary variable (yes or no).	Nealon J, Modin D, Ghosh RE, et al. The feasibility of pragmatic influenza vaccine randomized controlled real-world trials in Denmark and England. NPJ Vaccines 2022;7(1) doi: https://doi.org/10.1038/s41541-022-00444-6

	Likely mechanism: increased strain on heart to maintain blood pressure due to increased viscosity of blood		A range cardiovascular disease CPRD Aurum specific code lists, mapped to ICD-10 categories are available. To capture all cardiomyopathy cases the following code lists will be used in combination:<br>• *Ischaemic cardiomyopathy (ICD-10: I25)*<br>• *Cardiomyopathy (ICD-10: I42*
Cardiac arrest	High temperatures can increase risk of death for those who have previous experienced cardiac arrest<br><br>*Likely mechanism:* increased strain on heart to maintain blood pressure due to increased viscosity of blood	Diagnosis/record of cardiac arrest within the two years before death. Binary variable (yes or no).	Nealon J, Modin D, Ghosh RE, et al. The feasibility of pragmatic influenza vaccine randomized controlled real-world trials in Denmark and England. NPJ Vaccines 2022;7(1) doi: https://doi.org/10.1038/s41541-022-00444-6<br><br>A range cardiovascular disease CPRD Aurum specific code lists, mapped to ICD-10 categories are available. To capture cardiac arrest cases the following code lists will be used:<br>• *Cardiac arrest (ICD-10: I46)*
Heart failure	High temperatures increase the risk of mortality for individuals with history of heart failure<br><br>*Likely mechanism:* increased strain on heart to maintain blood pressure due to increased viscosity of blood	Diagnosis/record of heart failure within the two years before death. Binary variable (yes or no).	Nealon J, Modin D, Ghosh RE, et al. The feasibility of pragmatic influenza vaccine randomized controlled real-world trials in Denmark and England. NPJ Vaccines 2022;7(1) doi: https://doi.org/10.1038/s41541-022-00444-6<br><br>A range cardiovascular disease CPRD Aurum specific code lists, mapped to ICD-10 categories are available. To capture heart failure cases the following code lists will be used:<br>• *Heart failure (ICD-10: I50)*
Haemorrhage	High temperatures increase the risk of death for individuals who have previously suffered from bleeding in or around the brain.<br><br>*Likely mechanism:* blood flow redirected to where heat can escape, increasing potential for burst vessels and bleeding in or around the brain.	Diagnosis/record of haemorrhage within the two years before death. Binary variable (yes or no).	Nealon J, Modin D, Ghosh RE, et al. The feasibility of pragmatic influenza vaccine randomized controlled real-world trials in Denmark and England. NPJ Vaccines 2022;7(1) doi: https://doi.org/10.1038/s41541-022-00444-6<br><br>A range cardiovascular disease CPRD Aurum specific code lists, mapped to ICD-10 categories are available. To capture all haemorrhage cases the following code lists will be used in combination:<br>• *Subarachnoid haemorrhage (ICD-10: I60)*

			• *Intracerebral haemorrhage (ICD-10: I61)*<br>• *Other nontraumatic intracranial haemorrhage (ICD-10: I62)*
Stroke	Individuals with a history of stroke are at increased risk of mortality during periods of heat.<br><br>*Likely mechanism:* disruption in supply of blood/oxygen to brain cells for range of reasons that the CV system may be affected during heat events.	Diagnosis/record of stroke within the two years before death. Binary variable (yes or no).	Nealon J, Modin D, Ghosh RE, et al. The feasibility of pragmatic influenza vaccine randomized controlled real-world trials in Denmark and England. NPJ Vaccines 2022;7(1) doi: https://doi.org/10.1038/s41541-022-00444-6<br><br>A range cardiovascular disease CPRD Aurum specific code lists, mapped to ICD-10 categories are available. To capture all stroke cases the following code lists will be used in combination:<br>• *Cerebral infarction (ICD-10: I63)*<br>• *Stroke, not specified as haemorrhage or infarction (ICD-10: I64)*
Other cerebrovascular diseases	Individuals who suffer from blockages or narrowing of blood vessels/arteries are at increased risk of death during periods of high temperature.<br><br>*Likely mechanism:* disruption in supply of blood/oxygen to brain cells for range of reasons that the CV system may be affected during heat events.	Diagnosis/record of other cerebrovascular diseases within the two years before death. Binary variable (yes or no).	Nealon J, Modin D, Ghosh RE, et al. The feasibility of pragmatic influenza vaccine randomized controlled real-world trials in Denmark and England. NPJ Vaccines 2022;7(1) doi: https://doi.org/10.1038/s41541-022-00444-6<br><br>A range cardiovascular disease CPRD Aurum specific code lists, mapped to ICD-10 categories are available. To capture all other cerebrovascular disease cases the following code lists will be used in combination:<br>• *Occlusion and stenosis of precerebral arteries, not resulting in cerebral infarction (ICD-10: I65)*<br>• *Occlusion and stenosis of cerebral arteries, not resulting in cerebral infarction (ICD-10: I66)*<br>• *Other cerebrovascular diseases (ICD-10: 67)*<br>• *Cerebrovascular disorders in diseases classified elsewhere (ICD-10: I68)*
Arrythmia	Individuals who suffer from irregular heart beating are at increased risk of	Diagnosis/record of irregular heart beat within the two years before death. Binary variable (yes or no).	Nealon J, Modin D, Ghosh RE, et al. The feasibility of pragmatic influenza vaccine randomized controlled real-world trials in

	mortality during periods of high temperature.<br><br>*Likely mechanism:* additional strain on circulatory system may be too much for heart which is already beating irregularly.		Denmark and England. NPJ Vaccines 2022;7(1) doi: https://doi.org/10.1038/s41541-022-00444-6<br><br>A range cardiovascular disease CPRD Aurum specific code lists, mapped to ICD-10 categories are available. To capture all other cerebrovascular disease cases the following code lists will be used in combination:<br>• *Atrial fibrillation and flutter (ICD-10: I48)*<br>• *Other cardiac arrhythmias (ICD-10: I49)*
Emphysema	Individuals suffering from emphysema are at increased risk of mortality during periods of heat.<br><br>*Likely mechanism:* reduced ability to get additional oxygen to cells required when the body is overheating (e.g. heart muscle working harder to heat loss).	Diagnosis of emphysema within the two years before death. Binary variable (yes or no).	Nealon J, Modin D, Ghosh RE, et al. The feasibility of pragmatic influenza vaccine randomized controlled real-world trials in Denmark and England. NPJ Vaccines 2022;7(1) doi: https://doi.org/10.1038/s41541-022-00444-6<br><br>A range respiratory disease CPRD Aurum specific code lists, mapped to ICD-10 categories are available. The following code lists will be used:<br>• *Emphysema (ICD-10: J43)*
COPD	Individuals suffering from COPD are at increased risk of mortality during periods of heat.<br><br>*Likely mechanism:* reduced ability to get additional oxygen to cells required when the body is overheating (e.g. heart muscle working harder to heat loss).	Diagnosis of COPD within the two years before death. Binary variable (yes or no).	Nealon J, Modin D, Ghosh RE, et al. The feasibility of pragmatic influenza vaccine randomized controlled real-world trials in Denmark and England. NPJ Vaccines 2022;7(1) doi: https://doi.org/10.1038/s41541-022-00444-6<br><br>A range respiratory disease CPRD Aurum specific code lists, mapped to ICD-10 categories are available. The following code lists will be used:<br>• *Other chronic obstructive pulmonary disease (ICD-10: J44)*
Asthma	Individuals who suffer from asthma are at increased risk of death during high temperatures.	Diagnosis of asthma within the two years before death. Binary variable (yes or no).	Nealon J, Modin D, Ghosh RE, et al. The feasibility of pragmatic influenza vaccine randomized controlled real-world trials in Denmark and England. NPJ Vaccines 2022;7(1) doi: https://doi.org/10.1038/s41541-022-00444-6

	Likely mechanism: reduced ability to get additional oxygen to cells required when the body is overheating (e.g. heart muscle working harder to heat loss).		A range respiratory disease CPRD Aurum specific code lists, mapped to ICD-10 categories are available. The following code lists will be used:<br>• *Asthma (ICD-10: J45)*
Severe mental illness	Individuals who suffer from severe mental health disorders are at increased risk of mortality during periods of high temperatures.<br><br>*Likely mechanism:* Unclear, however it may be partly due to medication prescribed to control symptoms; inability of the individual to adapt their own behaviours and or environments; an inability of the individual to perceive a risk; combination of all three.	Definition used includes range of specific conditions, where the diagnosis suggests it is severe (e.g. severe psychosis, moderate and severe schizophrenic episode, severe depression, bipolar – severe manic episode etc). record of the above within two years prior to death.<br><br>Binary variable (yes or no).	Davidson, J and Strongman, H. Clinical codelist - CPRD Aurum - severe mental illness [Internet]. London School of Hygiene & Tropical Medicine; 2022. Available from: https://doi.org/10.17037/DATA.00002826.
Learning disability	Individuals with learning disabilities have a higher risk of mortality during periods of heat.<br><br>*Likely mechanism:* Unclear, however it may be partly due to inability of the individual to adapt their own behaviours and or environments; an inability of the individual to perceive a risk in the first place; or a combination of both.	Definition includes a wide range of learning disabilities, ranging from mild to severe general learning disabilities, and more specific syndromes which are not categorised.<br><br>Binary variable (yes or no).	Davidson, J, Warren-Gash, C and Cadogan, S. Clinical codelist - learning disabilities [Internet]. London School of Hygiene & Tropical Medicine; 2021. Available from: https://doi.org/10.17037/DATA.00002401.
Chronic Kidney disease	Individuals suffering from chronic kidney disease are at increased risk of mortality during periods of high temperatures.<br><br>*Likely mechanism:* dehydration as a mechanism to serious kidney injury –	Definition includes a range of terms linked to chronic kidney disease (e.g. all renal failure terms, hemofiltration therapy, glomerulonephritis etc).<br><br>Binary variable (yes or no)	Davidson, J, Warren-Gash, C, Mcdonald, H, Evans, D and Clay, S. Clinical codelist - chronic kidney disease [Internet]. London School of Hygiene & Tropical Medicine; 2021. Available from: https://doi.org/10.17037/DATA.00002406.

	reduced kidney function due to reduced water contend in blood.		
Psychosis	Individuals who suffer from psychosis are at increased risk of mortality during periods of high temperatures.<br><br>*Likely mechanism:* Unclear, however it may be partly due to medication prescribed to control symptoms; inability of the individual to adapt their own behaviours and or environments; an inability of the individual to perceive a risk; combination of all three.	Psychosis is quite a broad term, and as such this definition includes a wide range of mental health conditions which include delirium, psychosis etc. Due to the terms included in the list generation, it is not possible to comment on severity of psychosis/psychotic episodes.<br><br>Binary variable (yes or no)	Bespoke code list generated for this project by Daniel Omoyeni and Ross Thompson, clinically validated by Luis Baptista Mieiro<br><br>List available on GitHub here:<br>https://github.com/Rossdud/Clinical-code-lists
Anxiety	Individuals who suffer from anxiety are at increased risk of mortality during periods of high temperatures.<br><br>*Likely mechanism:* Unclear, however it may be partly due to medication prescribed to control symptoms; inability of the individual to adapt their own behaviours and or environments; an inability of the individual to perceive a risk; combination of all three.	Defining anxiety (and most mental health diseases) is challenging, due to the nature of primary care data. Terms included within the definition for anxiety were exhaustive and include any mention of anxiety conditions regardless of severity, where anxiety is mentioned. Refusal of questionnaires and family history were removed.<br><br>Binary variable (yes or no)	Bespoke code list generated for this project by Daniel Omoyeni and Ross Thompson, clinically validated by Luis Baptista Mieiro<br><br>List available on GitHub here:<br>https://github.com/Rossdud/Clinical-code-lists
Depression	Individuals who suffer from depression are at increased risk of mortality during periods of high temperatures.<br><br>*Likely mechanism:* Unclear, however it may be partly due to medication prescribed to control symptoms; inability of the individual to adapt their own behaviours and or environments; an inability of the individual to perceive a risk; combination of all three.	Defining depression (and most mental health diseases) is challenging, due to the nature of primary care data. Terms included within the definition for depression were exhaustive and include any mention of depression (excluding bi-polar) conditions regardless of severity. Refusal of questionnaires and family history were also removed.<br><br>Binary variable (yes or no)	Bespoke code list generated for this project by Daniel Omoyeni and Ross Thompson, clinically validated by Luis Baptista Mieiro<br><br>List available on GitHub here:<br>https://github.com/Rossdud/Clinical-code-lists

Bipolar disorder	Individuals who suffer from bipolar disorder are at increased risk of mortality during periods of high temperatures.<br><br>*Likely mechanism:* Unclear, however it may be partly due to medication prescribed to control symptoms; inability of the individual to adapt their own behaviours and or environments; an inability of the individual to perceive a risk; combination of all three.	Defining bipolar (and most mental health diseases) is challenging, due to the nature of primary care data. Terms included within the definition for bipolar disorder were exhaustive and include any mention of bipolar disorder regardless of severity. Refusal of questionnaires and family history among other routinely used terms not associated with a diagnosis were also removed.<br><br>Binary variable (yes or no)	Bespoke code list generated for this project by Daniel Omoyeni and Ross Thompson, clinically validated by Luis Baptista Mieiro<br><br>List available on GitHub here:<br>https://github.com/Rossdud/Clinical-code-lists
Schizophrenia	Individuals who suffer from schizophrenia are at increased risk of mortality during periods of high temperatures.<br><br>*Likely mechanism:* Unclear, however it may be partly due to medication prescribed to control symptoms; inability of the individual to adapt their own behaviours and or environments; an inability of the individual to perceive risk; combination of all three.	Variable is defined as any terms as recorded within primary care observations which include schizophrenia. From terms included within primary care data it is unclear of severity of symptoms is recorded in primary care data, therefore this will be a binary variable (yes or no)	Bespoke code list generated for this project by Daniel Omoyeni and Ross Thompson, clinically validated by Luis Baptista Mieiro<br><br>List available on GitHub here:<br>https://github.com/Rossdud/Clinical-code-lists
Dementia and Alzheimer's disease	Individuals who suffer from Alzheimer's and dementia are at increased risk of mortality during periods of high temperatures.<br><br>*Likely mechanism:* Unclear, however it may be partly due to medication prescribed to control symptoms; inability of the individual to adapt their own behaviours and or environments; an inability of the individual to perceive risk	All terms which indicate dementia and Alzheimer's diagnosis (all types and in association with other diseases).<br><br>Recording severity of diagnosis unlikely to be well recorded in primary care data.<br><br>Binary variable (yes or no)	Bespoke code list generated for this project by Daniel Omoyeni and Ross Thompson, clinically validated by Luis Baptista Mieiro<br><br>List available on GitHub here:<br>https://github.com/Rossdud/Clinical-code-lists

	on hot days; or a combination of all the above.		
Parkinson's disease	Individuals who suffer from Parkinson's disease are at increased risk of death during periods of high temperatures.<br><br>*Likely mechanism:* Unclear, but likely dehydration due to medication taken to control symptoms, as anti-Parkinson's medication are known to have a side effect of dehydration. Link this to Anti-Parkinson's medication variable (number 50)	All terms which indicate Parkinson's disease (including where it is in association with another disease)	Bespoke code list generated for this project by Daniel Omoyeni and Ross Thompson, clinically validated by Luis Baptista Mieiro<br><br>List available on GitHub here:<br>https://github.com/Rossdud/Clinical-code-lists
Hypothyroidism	Individuals with an underactive thyroid gland are at increased risk of death during periods of elevated temperatures.<br><br>*Likely mechanism:* body temperature rises because the basal metabolic rate is raised as there is increased oxygen consumption and the patients hypoactive adrenal function is globally reduced. While heat intolerance is most associated with hyperthyroidism, any thyroid disease, and particularly those related from autoimmune thyroid disfunction, can experience heat intolerance as the body struggles to maintain body temperature.	All terms related to hyperthyroidism/overactive thyroid.<br><br>Binary variable (yes or no)	Bespoke code list generated for this project by Daniel Omoyeni and Ross Thompson, clinically validated by Luis Baptista Mieiro<br><br>List available on GitHub here:<br>https://github.com/Rossdud/Clinical-code-lists
Hyperthyroidism	Individuals with an overactive thyroid gland are at increased risk of death during periods of elevated temperatures.<br><br>*Likely mechanism:* body temperature rises because the basal metabolic rate is	All terms related to hyperthyroidism/overactive thyroid.<br><br>Binary variable (yes or no)	Bespoke code list generated for this project by Daniel Omoyeni and Ross Thompson, clinically validated by Luis Baptista Mieiro<br><br>List available on GitHub here:<br>https://github.com/Rossdud/Clinical-code-lists

	raised as there is increased oxygen consumption and the patients hyperactive adrenal function is globally enhanced. The body will therefore have to work harder to lose excess heat potentially increasing strain on other organs sensitive to high temperatures		
Systolic blood pressure	Individuals with high SBP are at increased risk of death during heatwaves<br><br>*Likely mechanism*: increased strain on heart to maintain blood pressure due to increased viscosity of blood among other potential mechanisms related to CVD.	Terms included of relevance for extracting SBP measurements. Variable will then be classified according to NHS guidance.<br><br>Categorical variable:<br>• Low = <80 mmHg<br>• Normal = 80 to 120 mmHg<br>• Prehypertension = 120 to 139 mmHg<br>• HT stage 1 = 140 to 159 mmHg<br>HT Stage 2 = 160 mmHg <	Bespoke code list generated for this project by Daniel Omoyeni and Ross Thompson, clinically validated by Luis Baptista Mieiro<br><br>List available on GitHub here:<br>https://github.com/Rossdud/Clinical-code-lists
Diastolic blood pressure	Individuals with high DBP are at increased risk of death during heatwaves<br><br>*Likely mechanism*: increased strain on heart to maintain blood pressure due to increased viscosity of blood among other potential mechanisms related to CVD.	Terms included of relevance for extracting SBP measurements. Variable will then be classified according to NHS guidance.<br><br>Categorical variable:<br>• Low = <60 mmHg<br>• Normal = 60 to 80 mmHg<br>• PreHT = 80-89 mmHg<br>• HT Stage 1 = 90 to 99 mmHg<br>• HT Stage 2 = 100 mmHg <	Bespoke code list generated for this project by Daniel Omoyeni and Ross Thompson, clinically validated by Luis Baptista Mieiro<br><br>List available on GitHub here:<br>https://github.com/Rossdud/Clinical-code-lists
Cardiac Glycosides	Individuals taking cardiac glycosides to treat circulatory system conditions are at increased risk of death during heatwaves.	Defined initially by BNF chapter code (020101), then secondly by drug names of those identified within the BNF chapter. Formulation and concentration of prescribed medication is out of the scope of this project.	Bespoke code list generated for this project by Daniel Omoyeni and Ross Thompson, clinically validated by Luis Baptista Mieiro<br><br>List available on GitHub here:<br>https://github.com/Rossdud/Clinical-code-lists

	Likely mechanism: Unclear however the drug causes a more forceful heartbeat, therefore may increase workload of already strained muscle during periods of heat.	Binary variable (yes or no)	
Diuretics	Individuals taking diuretics are at increased risk of death during heatwaves.<br><br>Likely mechanism: Dehydration, as diuretics are designed to rid the body of sodium and water.	Defined initially by BNF chapter codes (020201, 020202, 020203, 020204, 020205, 020206), the secondly by drug names of those identified within the BNF chapters. Formulation and concentration of prescribed medication is out of the scope of this project.<br><br>Binary variable (yes or no)	Bespoke code list generated for this project by Daniel Omoyeni and Ross Thompson, clinically validated by Luis Baptista Mieiro<br><br>List available on GitHub here: https://github.com/Rossdud/Clinical-code-lists
Beta blockers	Individuals taking beta blockers are at increased risk of mortality during heatwaves.<br><br>Likely mechanism: interference of thermoregulatory mechanisms, making the heart work harder to get blood to the surface for the body to radiate excess heat.	Defined initially by BNF chapter codes (020400), the secondly by drug names of those identified within the BNF chapter. Formulation and concentration of prescribed medication is out of the scope of this project.<br><br>Binary variable (yes or no)	Bespoke code list generated for this project by Daniel Omoyeni and Ross Thompson, clinically validated by Luis Baptista Mieiro<br><br>List available on GitHub here: https://github.com/Rossdud/Clinical-code-lists
ACE inhibitors	As above, but specifically for ACE inhibitors. Ace inhibitors have been singled out specifically as they are mentioned within the literature in addition to the wider anti-hypertension drugs.	Defined initially by BNF chapter codes (020505), the secondly by drug names of those identified within the BNF chapter. Formulation and concentration of prescribed medication is out of the scope of this project. NOTE: this variable is ACE inhibitors only<br><br>Binary variable (yes or no)	Bespoke code list generated for this project by Daniel Omoyeni and Ross Thompson, clinically validated by Luis Baptista Mieiro<br><br>List available on GitHub here: https://github.com/Rossdud/Clinical-code-lists

Vasoconstrictor sympathomimetics	Individuals who are using vasoconstrictor medications to address low blood pressure or severe allergic reactions are at increased risk of death during heatwaves.<br><br>*Likely mechanism*: Vasoconstrictors increase blood pressure which can increase risk of stroke and other cardiovascular issues.	Defined initially by BNF chapter codes (020700), the secondly by drug names of those identified within the BNF chapter. Formulation and concentration of prescribed medication is out of the scope of this project.<br><br>Binary variable (yes or no)	Bespoke code list generated for this project by Daniel Omoyeni and Ross Thompson, clinically validated by Luis Baptista Mieiro<br><br>List available on GitHub here: https://github.com/Rossdud/Clinical-code-lists
Non-steroidal anti-inflammatory drugs	Individuals taking NSAIDs during heatwaves are at increased risk<br><br>*Likely mechanism*: NSAIDs are known to interfere with hormones involved in thermoregulation.	Defined initially by BNF chapter codes (100101), then secondly by drug names of those identified within the BNF chapter. Formulation and concentration of prescribed medication is out of the scope of this project.<br><br>Binary variable (yes or no)	Bespoke code list generated for this project by Daniel Omoyeni and Ross Thompson, clinically validated by Luis Baptista Mieiro<br><br>List available on GitHub here: https://github.com/Rossdud/Clinical-code-lists
Anticholinergic drugs	Individuals taking Anticholinergics during heatwaves are at increased risk<br><br>*Likely mechanism*: Anticholinergics are known to interfere with thermoregulation and potentially blood pressure	Presence of Anticholinergics as identified within clinical code list (previously available). Formulation and concentration of prescribed medication is out of the scope of this project.<br><br>Binary variable (yes or no)	Archer L, Koshiaris C, Lay-Flurrie S, et al. Development and external validation of a risk prediction model for falls in patients with an indication for antihypertensive treatment: retrospective cohort study. *BMJ* 2022;379:e070918. doi: 10.1136/bmj-2022-070918<br><br>CPRD Aurum specific code list generated for *the STRAtifying Treatments In the multi-morbid Frail elderlY (STRATIFY) study.*

Table S2. Description of exposure data series in analysis.

Variable	Observations	Proportion	Mean	Std. dev.	Min	Max
Temperature (°C) *(England)*	430,682	100.00%	15.7	3.3	5.7	27.7
London	65,145	26.33%	17.1	3.5	7.7	27.7
The South *(SW & SE)*	149,502	23.83%	15.9	3.1	6.7	25.6
Midlands and East *(WM, EM, EoE)*	102,630	15.13%	15.6	3.3	6.1	26.4
The North *(NE, NW & Y&H)*	113,405	34.71%	14.9	3.0	5.7	24.8
Ozone (ug/m^3) *(London)*	65,145	15.13%	48.9	16.1	11	126.7
NO_2 (ug/m^3) *(London)*	65,145	15.13%	21.9	9.8	3.5	61.4
PM_{10} (ug/m^3) *(London)*	65,145	15.13%	16.7	7.4	6	47.7

NW = Northwest; NE = Northeast; Y&H = Yorkshire and the Humber; WM = West Midlands; EM = East Midlands; EoE = East of England; Lon = London; SE = Southeast; SW = Southwest

Table S3 Temperature thresholds used in sub-national analysis derived from the temperature mortality relationships.

Sub-national area	MMT = RR=1	Low =RR-1.1
London	17	22
The South *(SW & SE)*	17	21.5
Midlands and East *(WM, EM, EoE)*	17	22
The North *(NE, NW & Y&H)*	16	21.5

NW = Northwest; NE = Northeast; Y&H = Yorkshire and the Humber; WM = West Midlands; EM = East Midlands; EoE = East of England; Lon = London; SE = Southeast; SW = Southwest

Table S4 Results for national and sub-national level unadjusted odds ratios for individuals with pre-existing conditions and prescribed medication with very strong to moderate evidence of increased risk of death during periods of heat using the low impact threshold compared to the minimum mortality temperature and the relative effect modification index (REM).

Variable	National		London		The South		Mids and East		The North	
	OR (95% CI)	REM	OR (95% CI)	REM	OR (95% CI)	REM	OR (95% CI)	REM	OR (95% CI)	REM
Whole population*	**1.09 (1.08 to 1.11)**	**1.00**	**1.09 (1.07 to 1.11)**	**1.00**	**1.09 (1.07 to 1.11)**	**1.00**	**1.07 (1.03 to 1.11)**	**1.00**	**1.17 (1.09 to 1.25)**	**1.00**
Diabetes	**1.12 (1.07 to 1.17)**	**1.02**	**1.09 (1.02 to 1.16)**	**1.00**	**1.14 (1.07 to 1.21)**	**1.05**	1.11 (0.99 to 1.25)	1.04	1.13 (0.93 to 1.37)	0.97
Heart Failure	**1.11 (1.04 to 1.19)**	**1.02**	**1.12 (1.01 to 1.23**	**1.02**	1.06 (0.96 to 1.16)	0.97	1.10 (0.94 to 1.28)	1.03	**1.41 (1.07 to 1.87)**	**1.21**
Haemorrhage	**1.25 (1.06 to 1.49)**	**1.15**	1.21 (0.95 to 1.55)	1.11	1.05 (0.83 to 1.33)	0.97	1.54 (0.93 to 2.48)	1.42	1.57 (0.78 to 3.16)	1.34
Stroke	**1.14 (1.03 to 1.25)**	**1.04**	1.11 (0.97 to 1.27)	1.02	1.11 (0.98 to 1.26)	1.02	1.16 (0.91 to 1.48)	1.09	1.41 (0.98 to 2.01)	1.20
Arrythmia	**1.09 (1.03 to 1.16)**	**1.00**	1.06 (0.97 to 1.16)	0.97	**1.09 (1.01 to 1.18)**	**1.00**	1.11 (0.97 to 1.28)	1.04	1.15 (0.91 to 1.44)	0.98
Occultation	1.35 (0.90 to 2.04)	1.24	0.99 (0.78 to 1.25)	0.91	1.06 (0.90 to 1.27)	0.98	**1.14 (1.00 to 2.00)**	**1.32**	0.88 (0.48 to 1.62)	0.76
COPD	**1.14 (1.02 to 1.27)**	**1.04**	**1.22 (1.04 to 1.43)**	**1.12**	1.04 (0.90 to 1.21)	0.96	1.18 (0.93 to 1.50)	1.10	0.98 (0.69 to 1.39)	0.84
Asthma	1.11 (0.99 to 1.24)	1.01	**1.17 (1.01 to 1.37)**	**1.08**	0.98 (0.84 to 1.14)	0.90	1.12 (0.86 to 1.45)	1.05	0.81 (0.50 to 1.31)	0.69
Severe Mental Health	**1.21 (1.01 to 1.45)**	**1.11**	1.12 (0.90 to 1.40)	1.03	**1.79 (1.30 to 2.47)**	**1.64**	1.24 (0.73 to 2.11)	1.16	0.35 (0.09 to 1.29)	0.30
Psychosis	**1.17 (1.03 to 1.32)**	**1.07**	1.20 (0.99 to 1.45)	1.10	1.06 (0.91 to 1.23)	0.97	1.23 (0.86 to 1.76)	1.15	1.39 (0.82 to 2.36)	1.19
Depression	**1.25 (1.09 to 1.44)**	**1.15**	**1.24 (1.04 to 1.49)**	**1.14**	1.14 (0.94 to 1.38)	1.05	1.27 (0.88 to 1.83)	1.19	1.36 (0.75 to 2.46)	1.16
Bipolar Disorder	1.03 (0.69 to 1.55)	0.94	0.54 (0.21 to 1.37)	0.49	**3.02 (1.63 to 5.60)**	**2.78**	-	-	-	-
Alzheimer's & Dementia	**1.09 (1.03 to 1.16)**	**1.00**	1.06 (0.98 to 1.15)	0.97	**1.11 (1.03 to 1.19)**	**1.02**	1.11 (0.98 to 1.27)	1.04	1.23 (0.96 to 1.58)	1.05
Parkinson's Disease	**1.22 (1.05 to 1.41)**	**1.11**	1.12 (0.91 to 1.39)	1.03	**1.27 (1.05 to 1.53)**	**1.16**	1.09 (0.74 to 1.60)	1.02	1.74 (0.78 to 3.91)	1.49
Chronic Kidney Disease	1.07 (0.99 to 1.15)	0.98	1.01 (0.89 to 1.09)	0.93	**1.11 (1.01 to 1.22)**	**1.02**	1.09 (0.92 to 1.28)	1.02	1.18 (0.91 to 1.53)	1.01
Cardiac Glycosides	**1.14 (1.03 to 1.26)**	**1.04**	1.07 (0.93 to 1.24)	0.98	**1.14 (1.02 to 1.29)**	**1.05**	**1.26 (1.00 to 1.60)**	**1.18**	1.49 (0.94 to 2.34)	1.27
Diuretics	**1.09 (1.04 to 1.13)**	**0.99**	**1.07 (1.01 to 1.14)**	**0.98**	**1.11 (1.06 to 1.17)**	**1.02**	1.06 (0.96 to 1.16)	0.99	1.16 (0.98 to 1.37)	0.99
Beta Blockers	**1.09 (1.05 to 1.14)**	**1.00**	1.05 (0.99 to 1.12)	0.97	**1.13 (1.07 to 1.19)**	**1.03**	1.08 (0.98 to 1.20)	1.02	**1.27 (1.06 to 1.51)**	**1.08**
Ace Inhibitors	**1.08 (1.04 to 1.12)**	**0.99**	**1.07 (1.01 to 1.13)**	**0.98**	**1.09 (1.03 to 1.15)**	**1.00**	1.05 (0.95 to 1.17)	0.99	**1.22 (1.03 to 1.45)**	**1.05**
Vasoconstrictors	**1.83 (1.19 to 2.80)**	**1.67**	-	-	**1.76 (1.00 to 3.09)**	**1.62**	-	-	-	-
NSAIDs	**1.13 (1.08 to 1.19)**	**1.03**	**1.11 (1.04 to 1.19)**	**1.02**	**1.14 (1.07 to 1.21)**	**1.04**	**1.16 (1.03 to 1.29)**	**1.08**	**1.31 (1.09 to 1.59)**	**1.12**

Bold values indicate estimates with moderate to very strong evidence ($p<0.05$) that individuals with a valid primary care record have an increased odds of death on hot days when a Low impact HHA is likely to be issued by UKHSA

REM is the relative effect modification index which is calculated by dividing the OR of the specific factor of interest by the reference factor, here taken as whole study sample population

Minimum mortality temperatures and low Impact temperature thresholds used in each sub-national areas analysis are provided in table S3 in supplemental materials

Table S5 OR estimates and 95% CI and p-values for all clinical individual-level risk factors investigated, for the whole population using both the Low Impact threshold (temperature associated with RR of 1.1) and Medium Impact threshold (RR of 1.2). In addition, the Relative Effect Modification index (REM) us also reported for each variable investigated.

**Variable**	**Low Impact threshold**			**Medium Impact threshold**		
	**OR (95% CI)**	**p-value**	**REM**	**OR (95% CI)**	**p-value**	**REM**
Whole population	1.09 (1.08 to 1.11)	<0.001	n/a	1.20 (1.16 to 1.25)	<0.001	n/a
Diastolic blood pressure						
Low DPB	1.07 (1.00 to 1.15)	0.053	0.96	1.12 (0.94 to 1.33)	0.195	0.91
Normal DBP	1.11 (1.08 to 1.14)	<0.001	1.00	1.23 (1.15 to 1.32)	<0.001	1.00
Prehypertensive	1.15 (1.1 0to 1.21)	<0.001	1.04	1.35 (1.21 to 1.51)	<0.001	1.10
Hypertension Stage 1	1.06 (0.96 to 1.16)	0.270	0.95	1.09 (0.87 to 1.38)	0.444	0.89
Hypertension Stage 2	1.09 (0.91 to 1.31)	0.349	0.98	1.07 (0.68 to 1.69)	0.766	0.87
Hypertension (all)	1.06 (0.98 to 1.15)	0.166	0.96	1.08 (0.88 to 1.33)	0.458	0.88
Systolic blood pressure						
Low SPB	1.21 (0.90 to 1.63)	0.208	1.07	1.20 (0.55 to 2.59)	0.652	0.92
Normal SBP	1.13 (1.09 to 1.17)	<0.001	1.00	1.30 (1.30 to 1.43)	<0.001	1.00
Prehypertensive	1.11 (1.03 to 3.16)	<0.001	0.98	1.24 (1.14 to 1.34)	<0.001	0.95
Hypertension Stage 1	1.08 (1.03 to 0.13)	0.003	0.95	1.14 (1.01 to 1.29)	0.035	0.88
Hypertension Stage 2	1.08 (0.97 to 1.20)	0.169	0.95	1.15 (0.87 to 1.52)	0.318	0.88
Hypertension (all)	1.08 (1.03 to 1.13)	0.001	0.95	1.14 (1.02 to 1.28)	0.021	0.88
Chronic conditions						
Diabetes	1.12 (1.07 to 1.17)	<0.001	1.02	1.25 (1.12 to 1.40)	<0.001	1.04
Myocardial infarction	1.04 (0.91 to 1.19)	0.593	0.95	1.15 (0.82 to 1.62)	0.412	0.96
Cardiomyopathy	0.87 (0.63 to 1.21)	0.414	0.80	0.65 (0.29 to 1.47)	0.302	0.54
Cardiac Arrest	1.01 (0.79 to 1.29)	0.919	0.93	0.84 (0.44 to 1.60)	0.597	0.70
Heart Failure	1.11 (1.04 to 1.19)	0.003	1.02	1.24 (1.04 to 1.47)	0.015	1.03
Haemorrhage	1.25 (1.06 to 1.49)	0.010	1.15	1.65 (1.08 to 2.54)	0.021	1.38
Stroke	1.14 (1.03 to 1.25)	0.010	1.04	1.20 (0.93 to 1.54)	0.166	0.99
Other CVD	0.96 (0.72 to 1.29)	0.809	0.88	0.71 (0.31 to 1.64)	0.427	0.59
Arrythmia	1.09 (1.03 to 1.16)	0.004	1.00	1.17 (1.00 to 1.36)	0.043	0.97
Occultation	1.35 (0.90 to 2.04)	0.147	1.24	1.13 (0.97 to 1.31)	0.118	0.94
Emphysema	1.00 (0.76 to 1.31)	0.983	0.91	1.25 (0.66 to 2.35)	0.492	1.04
CPOD	1.14 (1.02 to 1.27)	0.018	1.04	1.28 (0.97 to 1.69)	0.077	1.07
Asthma	1.11 (0.99 to 1.24)	0.070	1.01	1.13 (0.87 to 1.47)	0.353	0.94
Severe Mental Health	1.21 (1.01 to 1.45)	0.041	1.11	1.15 (0.73 to 1.81)	0.541	0.96
Learning disability	0.85 (0.62 to 1.17)	0.312	0.78	0.31 (0.10 to 1.03)	0.055	0.26
Psychosis	1.17 (1.03 to 1.32)	0.014	1.07	1.46 (1.08 to 1.98)	0.015	1.21
Anxiety	1.08 (0.97 to 1.20)	0.155	0.99	1.30 (0.98 to 1.73)	0.073	1.08
Depression	1.25 (1.09 to 1.44)	0.001	1.15	1.25 (0.89 to 1.75)	0.203	1.04
Bipolar Disorder	1.03 (0.69 to 1.55)	0.877	0.94	0.81 (0.21 to 3.17)	0.766	0.68
Schizophrenia	1.18 (0.91 to 1.54)	0.208	1.08	1.22 (0.64 to 2.34)	0.540	1.02
Alzheimer’s & Dementia	1.09 (1.03 to 1.16)	0.002	1.00	1.13 (0.97 to 1.30)	0.113	0.94

Parkinson's Disease	1.22 (1.05 to 1.41)	0.008	1.11	1.46 (1.02 to 2.08)	0.040	1.21
Hypothyroidism	0.99 (0.87 to 1.11)	0.812	0.90	1.09 (0.80 to 1.48)	0.575	0.91
Hyperthyroidism	1.01 (0.68 to 1.49)	0.965	0.92	1.69 (0.60 to 4.74)	0.319	1.41
Chronic Kidney Disease	1.07 (0.99 to 1.15)	0.071	0.98	1.08 (0.91 to 1.28)	0.387	0.90
Prescribed medications						
Cardiac Glycosides	1.14 (1.03 to 1.26)	0.011	1.04	1.14 (0.87 to 1.50)	0.348	0.95
Diuretics	1.09 (1.04 to 1.13)	<0.001	0.99	1.19 (1.07 to 1.32)	0.001	0.99
Beta Blockers	1.09 (1.05 to 1.14)	<0.001	1.00	1.21 (1.09 to 1.34)	<0.001	1.00
Ace Inhibitors	1.08 (1.04 to 1.12)	<0.001	0.99	1.17 (1.06 to 1.30)	0.002	0.97
Vasoconstrictors	1.83 (1.19 to 2.80)	0.006	1.67	6.94 (1.93 to 24.98)	0.003	5.77
NSAIDs	1.13 (1.08 to 1.19)	<0.001	1.03	1.27 (1.13 to 1.43)	<0.001	1.06
Anticholinergic drugs	1.07 (0.94 to 1.21)	0.316	0.97	0.94 (0.68 to 1.31)	0.723	0.78
